# Poly[tetra-μ-cyanido-dipyridine­cadmium(II)zinc(II)]

**DOI:** 10.1107/S1600536809054579

**Published:** 2010-01-09

**Authors:** Sheng Li, Kun Tang, Fu-Li Zhang

**Affiliations:** a College of Medicine, Henan University, Kaifeng 475003, People’s Republic of China

## Abstract

In the title coordination polymer, [CdZn(CN)_4_(C_5_H_5_N)_2_]_*n*_, the Zn^II^ atom (site symmetry 222) adopts a distorted ZnC_4_ tetra­hedral geometry, being coordinated by four crystallographically equivalent cyanide ions. The cyanide ion bridges to a Cd^II^ centre *via* its N atom. The Cd atom (site symmetry 2/*m*) coordination is a distorted CdN_6_ octa­hedron, arising from four cyanide N atoms and two pyridine N atoms. The complete pyridine mol­ecule is generated by *m* symmetry, with the N atom and one C atom lying on the reflecting plane. In the crystal, the bridging cyanide ions result in a three-dimensional network.

## Related literature

For background to cyanide-containing coordination networks, see: Vasylyev & Neumann (2006 ▶).
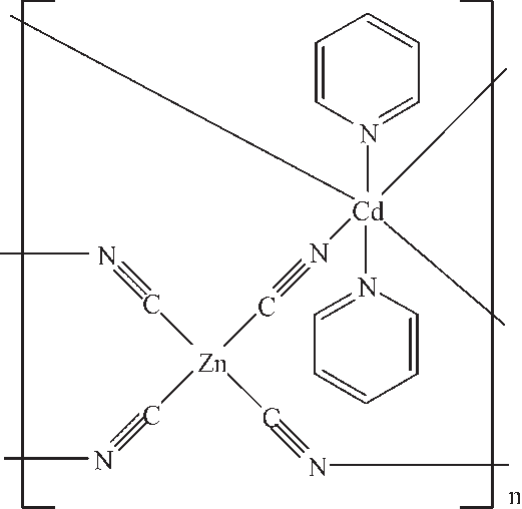


## Experimental

### 

#### Crystal data

[CdZn(CN)_4_(C_5_H_5_N)_2_]
*M* *_r_* = 440.05
Orthorhombic, 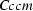

*a* = 9.514 (4) Å
*b* = 13.935 (6) Å
*c* = 13.965 (6) Å
*V* = 1851.5 (14) Å^3^
*Z* = 4
Mo *K*α radiation
μ = 2.45 mm^−1^
*T* = 296 K
0.44 × 0.28 × 0.22 mm

#### Data collection

Bruker APEXII CCD diffractometer
Absorption correction: multi-scan (*SADABS*; Bruker, 2001 ▶) *T*_min_ = 0.412, *T*_max_ = 0.615
4068 measured reflections
827 independent reflections
711 reflections with *I* > 2σ(*I*)
*R*_int_ = 0.051

#### Refinement

*R*[*F* ^2^ > 2σ(*F* ^2^)] = 0.050
*wR*(*F* ^2^) = 0.136
*S* = 1.00
827 reflections
57 parameters
H-atom parameters not refined
Δρ_max_ = 2.77 e Å^−3^
Δρ_min_ = −1.34 e Å^−3^

### 

Data collection: *APEX2* (Bruker, 2004 ▶); cell refinement: *SAINT-Plus* (Bruker, 2001 ▶); data reduction: *SAINT-Plus*; program(s) used to solve structure: *SHELXS97* (Sheldrick, 2008 ▶); program(s) used to refine structure: *SHELXL97* (Sheldrick, 2008 ▶); molecular graphics: *SHELXTL* (Sheldrick, 2008 ▶); software used to prepare material for publication: *SHELXTL*.

## Supplementary Material

Crystal structure: contains datablocks I, global. DOI: 10.1107/S1600536809054579/hb5184sup1.cif

Structure factors: contains datablocks I. DOI: 10.1107/S1600536809054579/hb5184Isup2.hkl

Additional supplementary materials:  crystallographic information; 3D view; checkCIF report

## Figures and Tables

**Table 1:** Selected bond lengths (Å)

Cd—N3	2.345 (4)
Cd—N1	2.354 (5)
Zn1—C4	2.037 (4)

**Fig. 1.**
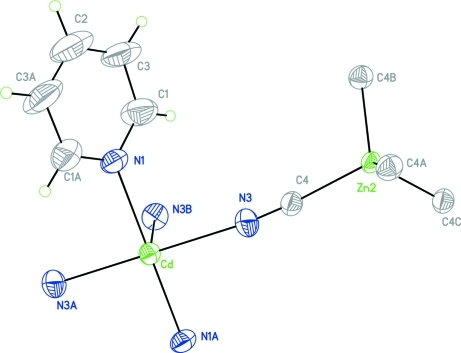
A view of (I) with the unique atom-labelling scheme. Displacement ellipsoids are drawn at the 30% probability level.

**Fig. 2.**
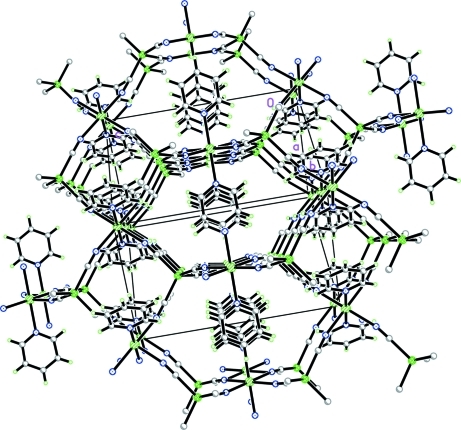
A view of (I) supramolecular strcuture.

## supplementary crystallographic information

### Comment

It has always been the interest of many chemists to design and synthesize novel metal cyano compounds which possess broad applications in host–guest chemistry, catalysis, photochemistry and electrical conductivity *etc* (Vasylyev & Neumann, 2006). Herein, we report a new crystal structure.

In the asymmetric unit of complex I, there exhibit one cyano ios, half pyridine, one Cd(II), and one Zn^II^, figure 1. The Zn^II^ ion surrounded by four cyano^-1^ ligands is tetra-coordinated by four C atoms, with tetrahedral coordination sphere. The bond distances of Zn—C is 2.037 (4) /%A in the normal range compared to the reported complexes containing the Zn—C—N—Cd atoms (Vasylyev & Neumann, 2006). The cadmium(II) is hexacoordianted by six N atoms from four cyano ions and two pyridine, located in the center of the coordinated octahedral geometry. It is worthy noting that the complex exhibits three-dimensional structure *via* the bridge of cyano ions, figure 2.

### Experimental

The starting materials of sodium cyanide (0.049 g, 1 mmol) and ZnSO_4_.7H_2_O (0.07 g, 0.25 mmol), and CdSO_4_ (0.05 g, 0.25 mmol) were refluxed in the mixture solution (CH_3_OH: pyridine = 10:1) until all solid was dissolved. The solution was cooled to room temperature and filtered.  Colourless blocks of (I) were obtained by allowing slow evaporation.

### Refinement

All hydrogen atoms bound to carbon were refined using a riding model with distance C—H = 0.93 Å, *U*_iso_ = 1.2*U*_eq_ (C) for aromatic atoms.

### Crystal data

**Table:**

[CdZn(CN)_4_(C_5_H_5_N)_2_]	*F*(000) = 856
*M**_r_* = 440.05	*D*_x_ = 1.579 Mg m^−^^3^
Orthorhombic, *C**c**c**m*	Mo *K*α radiation, λ = 0.71073 Å
Hall symbol: -C 2 2c	Cell parameters from 2697 reflections
*a* = 9.514 (4) Å	θ = 2.6–27.9°
*b* = 13.935 (6) Å	µ = 2.45 mm^−^^1^
*c* = 13.965 (6) Å	*T* = 296 K
*V* = 1851.5 (14) Å^3^	Block, colourless
*Z* = 4	0.44 × 0.28 × 0.22 mm

### Data collection

**Table:**

Bruker APEXII CCD diffractometer	827 independent reflections
Radiation source: fine-focus sealed tube	711 reflections with *I* > 2σ(*I*)
graphite	*R*_int_ = 0.051
φ and ω scans	θ_max_ = 25.0°, θ_min_ = 2.9°
Absorption correction: multi-scan (*SADABS*; Bruker, 2001)	*h* = −11→11
*T*_min_ = 0.412, *T*_max_ = 0.615	*k* = −16→8
4068 measured reflections	*l* = −16→15

### Refinement

**Table:**

Refinement on *F*^2^	Primary atom site location: structure-invariant direct methods
Least-squares matrix: full	Secondary atom site location: difference Fourier map
*R*[*F*^2^ > 2σ(*F*^2^)] = 0.050	Hydrogen site location: inferred from neighbouring sites
*wR*(*F*^2^) = 0.136	H-atom parameters not refined
*S* = 1.00	*w* = 1/[σ^2^(*F*_o_^2^) + (0.115*P*)^2^] where *P* = (*F*_o_^2^ + 2*F*_c_^2^)/3
827 reflections	(Δ/σ)_max_ = 0.001
57 parameters	Δρ_max_ = 2.77 e Å^−^^3^
0 restraints	Δρ_min_ = −1.34 e Å^−^^3^

### Special details

**Table:**

Geometry. All e.s.d.'s (except the e.s.d. in the dihedral angle between two l.s. planes) are estimated using the full covariance matrix. The cell e.s.d.'s are taken into account individually in the estimation of e.s.d.'s in distances, angles and torsion angles; correlations between e.s.d.'s in cell parameters are only used when they are defined by crystal symmetry. An approximate (isotropic) treatment of cell e.s.d.'s is used for estimating e.s.d.'s involving l.s. planes.
Refinement. Refinement of *F*^2^ against ALL reflections. The weighted *R*-factor *wR* and goodness of fit *S* are based on *F*^2^, conventional *R*-factors *R* are based on *F*, with *F* set to zero for negative *F*^2^. The threshold expression of *F*^2^ > σ(*F*^2^) is used only for calculating *R*-factors(gt) *etc*. and is not relevant to the choice of reflections for refinement. *R*-factors based on *F*^2^ are statistically about twice as large as those based on *F*, and *R*- factors based on ALL data will be even larger.

### Fractional atomic coordinates and isotropic or equivalent isotropic displacement parameters (Å^2^)

**Table:**

	*x*	*y*	*z*	*U*_iso_*/*U*_eq_	
Cd	1.2500	0.2500	0.5000	0.04119 (16)	
Zn1	1.0000	0.5000	0.2500	0.0357 (2)	
C4	1.1171 (4)	0.4109 (3)	0.3337 (3)	0.0495 (9)	
N3	1.1750 (4)	0.3577 (3)	0.3815 (3)	0.0643 (10)	
N1	1.0252 (5)	0.1795 (4)	0.5000	0.0592 (13)	
C1	0.9588 (8)	0.1595 (5)	0.4210 (4)	0.104 (2)	
H1A	1.0043	0.1715	0.3633	0.125*	
C2	0.7615 (11)	0.1001 (9)	0.5000	0.131 (4)	
H2A	0.6741	0.0703	0.5000	0.157*	
C3	0.8239 (8)	0.1213 (6)	0.4180 (6)	0.132 (3)	
H3A	0.7787	0.1109	0.3598	0.158*	

### Atomic displacement parameters (Å^2^)

**Table:**

	*U*^11^	*U*^22^	*U*^33^	*U*^12^	*U*^13^	*U*^23^
Cd	0.0414 (3)	0.0350 (3)	0.0472 (3)	0.0062 (2)	0.000	0.000
Zn1	0.0399 (4)	0.0276 (4)	0.0396 (4)	0.000	0.000	0.000
C4	0.051 (2)	0.0422 (18)	0.0553 (18)	−0.0008 (18)	−0.0004 (16)	0.0053 (16)
N3	0.069 (3)	0.055 (2)	0.069 (2)	0.010 (2)	−0.0084 (17)	0.0112 (17)
N1	0.050 (3)	0.052 (3)	0.076 (3)	−0.010 (2)	0.000	0.000
C1	0.101 (4)	0.124 (5)	0.087 (4)	−0.045 (4)	−0.003 (3)	−0.020 (3)
C2	0.086 (6)	0.105 (7)	0.201 (12)	−0.048 (5)	0.000	0.000
C3	0.115 (5)	0.147 (7)	0.133 (6)	−0.083 (5)	−0.026 (4)	−0.003 (5)

### Geometric parameters (Å, °)

**Table:**

Cd—N3^i^	2.345 (4)	C4—N3	1.140 (5)
Cd—N3^ii^	2.345 (4)	N1—C1	1.301 (6)
Cd—N3^iii^	2.345 (4)	N1—C1^iii^	1.301 (6)
Cd—N3	2.345 (4)	C1—C3	1.390 (10)
Cd—N1	2.354 (5)	C1—H1A	0.9300
Cd—N1^ii^	2.354 (5)	C2—C3^iii^	1.324 (9)
Zn1—C4^iv^	2.037 (4)	C2—C3	1.324 (9)
Zn1—C4	2.037 (4)	C2—H2A	0.9300
Zn1—C4^v^	2.037 (4)	C3—H3A	0.9300
Zn1—C4^vi^	2.037 (4)		
			
N3^i^—Cd—N3^ii^	89.74 (19)	C4^iv^—Zn1—C4^vi^	113.7 (2)
N3^i^—Cd—N3^iii^	180.0	C4—Zn1—C4^vi^	109.9 (2)
N3^ii^—Cd—N3^iii^	90.26 (19)	C4^v^—Zn1—C4^vi^	104.9 (2)
N3^i^—Cd—N3	90.26 (18)	N3—C4—Zn1	175.6 (4)
N3^ii^—Cd—N3	180.0	C4—N3—Cd	167.6 (3)
N3^iii^—Cd—N3	89.74 (19)	C1—N1—C1^iii^	116.0 (7)
N3^i^—Cd—N1	90.54 (14)	C1—N1—Cd	122.0 (3)
N3^ii^—Cd—N1	90.54 (14)	C1^iii^—N1—Cd	122.0 (3)
N3^iii^—Cd—N1	89.46 (14)	N1—C1—C3	123.8 (6)
N3—Cd—N1	89.46 (14)	N1—C1—H1A	118.1
N3^i^—Cd—N1^ii^	89.46 (14)	C3—C1—H1A	118.1
N3^ii^—Cd—N1^ii^	89.46 (14)	C3^iii^—C2—C3	119.9 (10)
N3^iii^—Cd—N1^ii^	90.54 (14)	C3^iii^—C2—H2A	120.1
N3—Cd—N1^ii^	90.54 (14)	C3—C2—H2A	120.1
N1—Cd—N1^ii^	180.0	C2—C3—C1	118.2 (7)
C4^iv^—Zn1—C4	104.9 (2)	C2—C3—H3A	120.9
C4^iv^—Zn1—C4^v^	109.9 (2)	C1—C3—H3A	120.9
C4—Zn1—C4^v^	113.7 (2)		

Symmetry codes: (i) −*x*+5/2, −*y*+1/2, *z*; (ii) −*x*+5/2, −*y*+1/2, −*z*+1; (iii) *x*, *y*, −*z*+1; (iv) −*x*+2, *y*, −*z*+1/2; (v) *x*, −*y*+1, −*z*+1/2; (vi) −*x*+2, −*y*+1, *z*.
